# Dosage balance acts as a time-dependent selective barrier to subfunctionalization

**DOI:** 10.1186/s12862-023-02116-y

**Published:** 2023-05-03

**Authors:** Amanda E. Wilson, David A. Liberles

**Affiliations:** Department of Biology and Center for Computational Genetics and Genomics, Temple University, Philadelphia, PA 19122 USA

**Keywords:** Whole genome duplication, Subfunctionalization, Dosage balance, Stochastic process model, Biophysical model, Genome content

## Abstract

**Background:**

Gene duplication is an important process for genome expansion, sometimes allowing for new gene functions to develop. Duplicate genes can be retained through multiple processes, either for intermediate periods of time through processes such as dosage balance, or over extended periods of time through processes such as subfunctionalization and neofunctionalization.

**Results:**

Here, we built upon an existing subfunctionalization Markov model by incorporating dosage balance to describe the interplay between subfunctionalization and dosage balance to explore selective pressures on duplicate copies. Our model incorporates dosage balance using a biophysical framework that penalizes the fitness of genetic states with stoichiometrically imbalanced proteins. These imbalanced states cause increased concentrations of exposed hydrophobic surface areas, which cause deleterious mis-interactions. We draw comparison between our Subfunctionalization + Dosage-Balance Model (Sub + Dos) and the previous Subfunctionalization-Only (Sub-Only) Model. This comparison includes how the retention probabilities change over time, dependent upon the effective population size and the selective cost associated with spurious interaction of dosage-imbalanced partners. We show comparison between Sub-Only and Sub + Dos models for both whole-genome duplication and small-scale duplication events.

**Conclusion:**

These comparisons show that following whole-genome duplication, dosage balance serves as a time-dependent selective barrier to the subfunctionalization process, by causing an overall delay but ultimately leading to a larger portion of the genome retained through subfunctionalization. This higher percentage of the genome that is ultimately retained is caused by the alternative competing process, nonfunctionalization, being selectively blocked to a greater extent. In small-scale duplication, the reverse pattern is seen, where dosage balance drives faster rates of subfunctionalization, but ultimately leads to a smaller portion of the genome retained as duplicates. This faster rate of subfunctionalization is because the dosage balance of interacting gene products is negatively affected immediately after duplication and the loss of a duplicate restores the stoichiometric balance. Our findings provide support that the subfunctionalization of genes that are susceptible to dosage balance effects, such as proteins involved in complexes, is not a purely neutral process. With stronger selection against stoichiometrically imbalanced gene partners, the rates of subfunctionalization and nonfunctionalization slow; however, this ultimately leads to a greater proportion of subfunctionalized gene pairs.

**Supplementary Information:**

The online version contains supplementary material available at 10.1186/s12862-023-02116-y.

## Background

Gene duplication is a very important process in the evolution of genomes [1]. Many genes have undergone some type of duplication event in their history. The human lineage together with other vertebrates had two rounds of whole-genome duplication during the chordate-vertebrate transition [2, 3]. Plant phylogenies indicate many rounds of duplication events in their history [4–11]. Gene redundancy relaxes selection and makes faster evolutionary exploration of sequence space and gene function space possible [1, 12]. Duplicate copies may lead to the development of beneficial fitness effects, including novel morphological traits [13, 14]. To understand the evolutionary processes that follow gene duplication is to understand a source of genome expansion, pathway complexity, and functional innovation.

The process of gene duplication has been well studied in the past half century with two general categories of duplication events are small-scale duplication (SSD) events and large-scale events [15]. Small-scale duplication events affect relatively small sections of the genome. These errors usually occur during DNA replication or through transposition events [16]. Larger-scale duplication events include chromosomal or whole-genome duplication (WGD). These typically originate from non-disjunction events in meiosis and/or hybridization [17], and are typically rarer than their small-scale counterpart but can be beneficial [18, 19].

Genes that originate from whole-genome duplication and small-scale duplication events follow different patterns of retention [4, 20–24]. These different patterns occur because of their initial effect on the genome and cell function. Specifically, duplications and subsequent processes may affect the stoichiometric balance of gene products. Protein subunits often have a hydrophobic surface that are buried in a protein complex and function to aid in the binding of the complex. However, when subunits are not bound in their prospective protein complex, these hydrophobic surface areas are solvent exposed, so they will seek out a hydrophobic environment to bury into. The interactions caused by this force may lead to deleterious effects. Because duplicate copies often lead to higher expression, these copies can influence the stoichiometry of protein-complex subunits. Therefore, selection acts to maintain the stochiometric balance by removing expression of redundant copies to avoid these mis-interactions and aggregation of the gene products [25, 26].

Small-scale duplication events happen frequently, but they immediately interfere with the stoichiometric balance, so they tend not to be highly favorable, so selection favors the loss of these duplicated genes [23, 27, 28]. Large scale duplication events are more likely to include genes and their interacting partners, so there is selective pressure to keep the additional copies to avoid negatively affecting the stoichiometric balance [20, 29, 30]. Gene dosage balance describes the tendency for duplicated gene copies to be retained for an intermediate time immediately following the whole-genome duplication event [31–33]. This is thought to be why there is an initially fast gene loss rate of duplicate copies after small-scale duplication events, but a slower initial gene loss rate in larger duplication events, followed by a faster loss rate once the stoichiometric balance is affected [30, 33–42], unless this is counterbalanced by functional changes that affect fitness [33, 43, 44]. Fernández et al. [45] have also noted that beyond the increased waiting time for subfunctionalization enabled by dosage balance, that the two processes can interplay to affect fitness.

Nonfunctionalization, the process of making one of the copies of a gene no longer functional, is the most common fate of duplicated genes [1]. This is because there is a lot of opportunity to gain an early stop codon or some other loss of function mutation (for example, nonsense, frameshift), and the other copy can continue the ancestral function [39]. Of the genes that are ultimately retained for a long time, it is likely these extra copies have some sort of benefit or that they subfunctionalized; otherwise, probability dictates that random function degrading mutations would take over in a few million years [15, 46], either through neutral or selective processes. For duplicated copies that have generated a fitness advantage, there are a few generally accepted ways these gene copies can be beneficial. First, there is a small chance that it is beneficial to have extra copies of the same functionally exact gene as a way of upregulating that gene [47, 48], but that is likely not true for the vast majority of retained genes [49]. Other processes include regulatory neofunctionalization, coding function neofunctionalization, or specialization after subfunctionalization [1, 34, 50–52]. For genes that first subfunctionalize, it is also believed that subfunctionalization can be an intermediate step to ultimate neofunctionalization, in a process called subneofunctionalization [53, 54]. This is supported by the observation that duplicate genes that are retained over long evolutionary time periods do show patterns consistent with both neofunctionalization and subfunctionalization [55].

An important research question in the field is, “what makes some genes have large numbers of duplicates while other genes exclusively exist as singletons?”. One of the leading theories to explain this phenomenon is the gene duplicability hypothesis, that certain types of genes, whether that “type” is categorized by GO terms or by the complexity of its interaction network/pathway, are more likely to benefit from extra copies of these genes [56, 57]. This benefit may be because they may be more likely to gain new functions or it is favorable and mutationally accessible to specialize [46, 56, 58, 59]. Additionally, dosage balance may play a role in retention of the extra copies, especially depending on what kind of duplication event has occurred in genome history and when. Duplicated genes in dosage balance can also have effects on the expression of and interactions with other genes and their protein products across the genome. Such trans-acting effects can contribute to the selective landscape of the evolution of individual gene duplicate pairs in parallel to changes in the gene itself and its regulatory regions [60–64]

As previously suggested, as a whole-genome duplication event ages and is under the influence of gene dosage balance, the probability of retaining duplicate gene copies decreases [33, 34, 46, 57, 65]. Therefore, gene dosage balance is one important reason why retaining or losing duplicate gene copies after a whole-genome duplication event is a time-heterogeneous process. However, the dynamics and constraints of this process have not been fully explored. Previous attempts at modeling the effect that gene dosage balance has on duplicate gene copy retention were less mechanistic, utilizing survival analysis described by an increasing hazard function of duplicated and redundant gene copies [33, 34]. This type of model is a mathematical description of the observed phenomenon, it does not actually model the underlying biological process itself at the level of detail described here and therefore would not enable discovery of the effects of the process on the dynamics that are not obvious from data fitting.

Here, building upon an existing framework for subfunctionalization proposed by Force and Lynch [46] and developed as a full model by Stark et al. [59], we propose an alternative time-homogeneous model. This new model joins a chemical thermodynamic model with a population model to explore the selective pressures on the retention of duplicated genes through subfunctionalization when genes are influenced by dosage balance effects. Our model incorporates an element of fitness that depends on the stoichiometric balance. This fitness parameter models selective effects on gene duplicate retention through subfunctionalization. Subfunctionalization is typically conceptualized as a neutral process because each necessary function continues to be performed by one of the copies. We explored how losing functional expression of one of the copies affects the stoichiometric balance between that gene product and the other gene products. We expect that if a gene is sensitive to dosage balance effects, losing expression of one of the duplicate gene copies in a specific tissue or developmental stage will negatively affect the stoichiometric balance of gene products in that tissue or developmental stage, causing selection to act against both subfunctionalization and nonfunctionalization. We expect selection to act against any process that negatively affects the stoichiometric balance of gene products, including subfunctionalization because it would cause imbalance in specific expression domains, but even more so for nonfunctionalization because it affects the balance in all expression domains simultaneously. We built a modeling framework that produces this behavior by modeling the underlying process.

## Methods

### Calculating the sum concentration of exposed hydrophobic residues across expression domains

To ultimately model dosage balance, we are interested in estimating the magnitudinal effect that stoichiometric imbalance has on the fitness of each state of duplicate gene pairs. We model a heterodimer, with subunits A and B, which are transcribed and translated from Gene A (G_A_) and Gene B (G_B_) respectively. The heterodimer’s binding interface is formed by one binding site on each subunit, and the binding site consists of a patch of exposed hydrophobic residues. We expect there to be some concentration of unbound subunit A ([A]_free_), unbound subunit B ([B]_free_), as well as subunits A and B in their bound form as a heterodimer ([AB]) in a cell. The reaction that yields the bound form can be seen in Eq. 1. All parameter and variable symbols, definitions, and values are described in Table 2.

1 $${\text{A}} + {\text{ B}} \leftrightarrow {\text{AB}}$$

The equilibrium constant (*K*_eq_) for the reaction in Eq. 1 is based upon free energy differences. Quantitative data that is measurable for this kind of reaction include the *K*_eq_, the total concentration of the A subunit from the expression of G_A_ ([A]_total_), and total concentration of the B subunit from the expression of G_B_ ([B]_total_). We can use this quantifiable information to calculate [A]_free_, [B]_free_, [AB] and ultimately the concentration of patches of exposed hydrophobic residues ([hp]) (Eqs. 2, 3, and 4). The total concentration of a subunit is the sum of the concentration of subunits in the unbound form and the concentration of subunits in the bound form (Eqs. 3 and 4).

2 $$\left[ {{\text{hp}}} \right] = \left[ {\text{A}} \right]_{{{\text{free}}}} + \left[ {\text{B}} \right]_{{{\text{free}}}}$$

3 $$\left[ {\text{A}} \right]_{{{\text{total}}}} = \left[ {\text{A}} \right]_{{{\text{free}}}} + \left[ {{\text{AB}}} \right]$$

4 $$\left[ {\text{B}} \right]_{{{\text{total}}}} = \left[ {\text{B}} \right]_{{{\text{free}}}} + \left[ {{\text{AB}}} \right]$$

We can solve for the concentration of patches of exposed hydrophobic residues ([hp]) based on the above information by plugging in the [AB] solution above into the quadradic equation to get [A]_free_ and [B]_free_ in terms of *K*_eq_, [A]_total_, and [B]_total_, which are known (Eq. 5). Once we can solve for [AB] we can calculate [hp] for each regulatory domain.

5 $$[\mathrm{AB}] = \frac{({K}_{\mathrm{eq}}{[\mathrm{A}]}_{\mathrm{total}} + {K}_{\mathrm{eq}}{[\mathrm{B}]}_{\mathrm{total}} + 1) \pm \sqrt{(-{\left({K}_{\mathrm{eq}}{\left[\mathrm{A}\right]}_{\mathrm{total}} + {K}_{\mathrm{eq}}{\left[\mathrm{B}\right]}_{\mathrm{total}}+ 1\right)}^{2} -4{K}_{\mathrm{eq}}({K}_{\mathrm{eq}} * {[\mathrm{A}]}_{\mathrm{total}} * {[\mathrm{B}]}_{\mathrm{total}})})}{2{K}_{\mathrm{eq}}}$$

The conceptualize the stoichiometric imbalance “load” to be the sum of [hp] across each regulatory domain. Therefore, we assume the fitness of each state is inversely proportional to the sum of hydrophobic patches across *z* regulatory domains. Because of this relationship, we calculate fitness (*f*) using an inverse function, with the relationship between the sum of hydrophobic patches per cell and the corresponding fitness penalty scaled by *w* (Eq. 6).

6 $$f=\frac{1}{(1+w\sum_{1\to z}{\left[\mathrm{hp}\right]}_{z})}$$

Loss of expression of duplicate copies causes stoichiometric imbalance. The imbalance of gene products introduces a fitness consequence. We want to use see how the fitness consequences affects the probability of fixing such a loss mutation in a population. We use an existing framework to calculate the probability of fixing mutations (*g*) [66], plugging Eq. 6 into the fitness terms, with the relative fitness of the current state being *f*_*i*_ and the relative fitness of the next possible state being *f*_*j*_ and *N*_e_ being the effective population size (Eq. 7). We will use this calculation to calculate the rates between states in our Markov chain in the next section.

7 $$g= \frac{1- \frac{{f}_{i}}{{f}_{j}}}{1-{\frac{{f}_{i}}{{f}_{j}}}^{{N}_{\mathrm{e}}}}= \frac{1- \frac{{1+(w\sum_{1\to z}{[\mathrm{hp}]}_{z})}_{j}}{{1+(w\sum_{1\to z}{[\mathrm{hp}]}_{z})}_{i}}}{1-{\frac{{1+(w\sum_{1\to z}{[\mathrm{hp}]}_{z})}_{j}}{{1+(w\sum_{1\to z}{[\mathrm{hp}]}_{z})}_{i}}}^{{N}_{\mathrm{e}}}}$$

### Continuous-time Markov chain

**Fig. 1:**
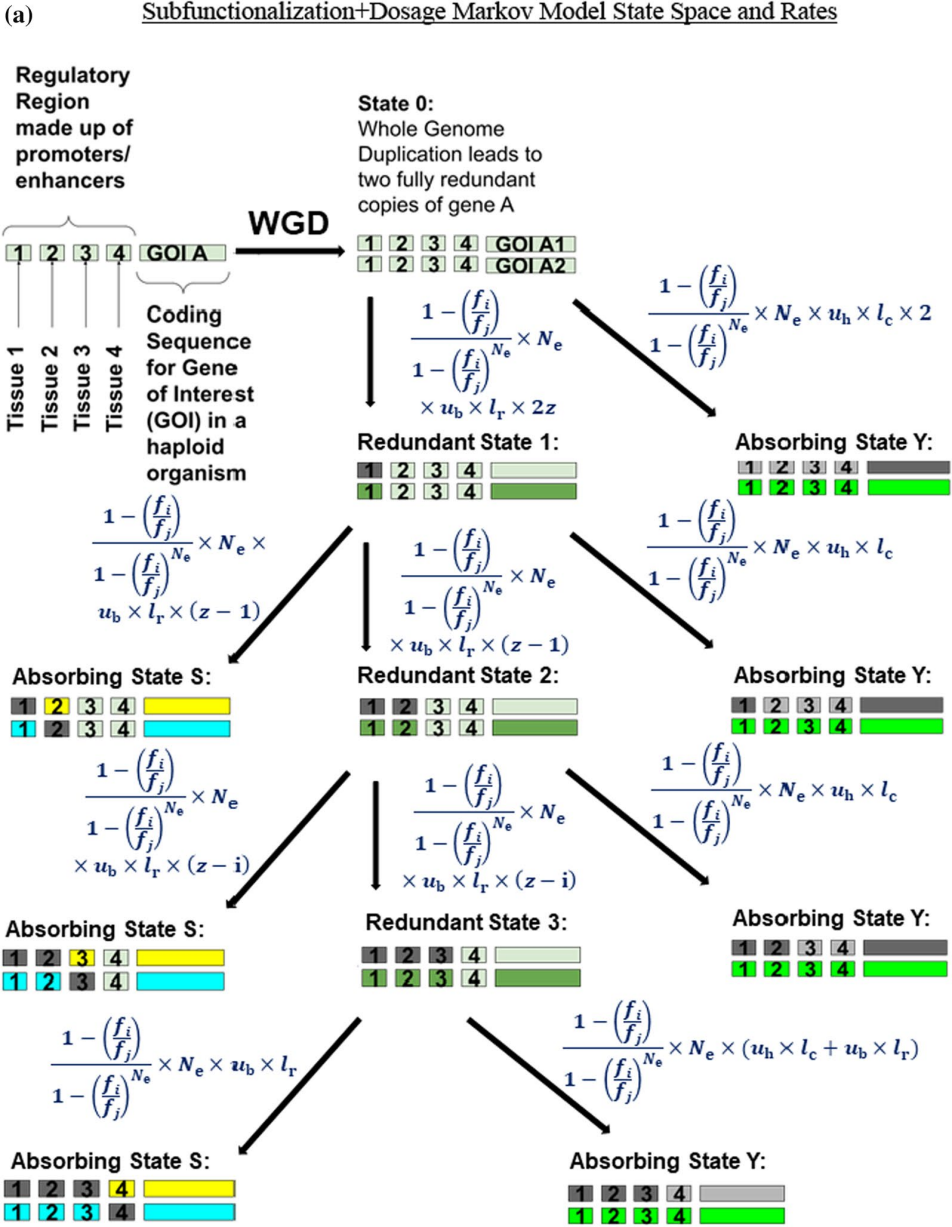

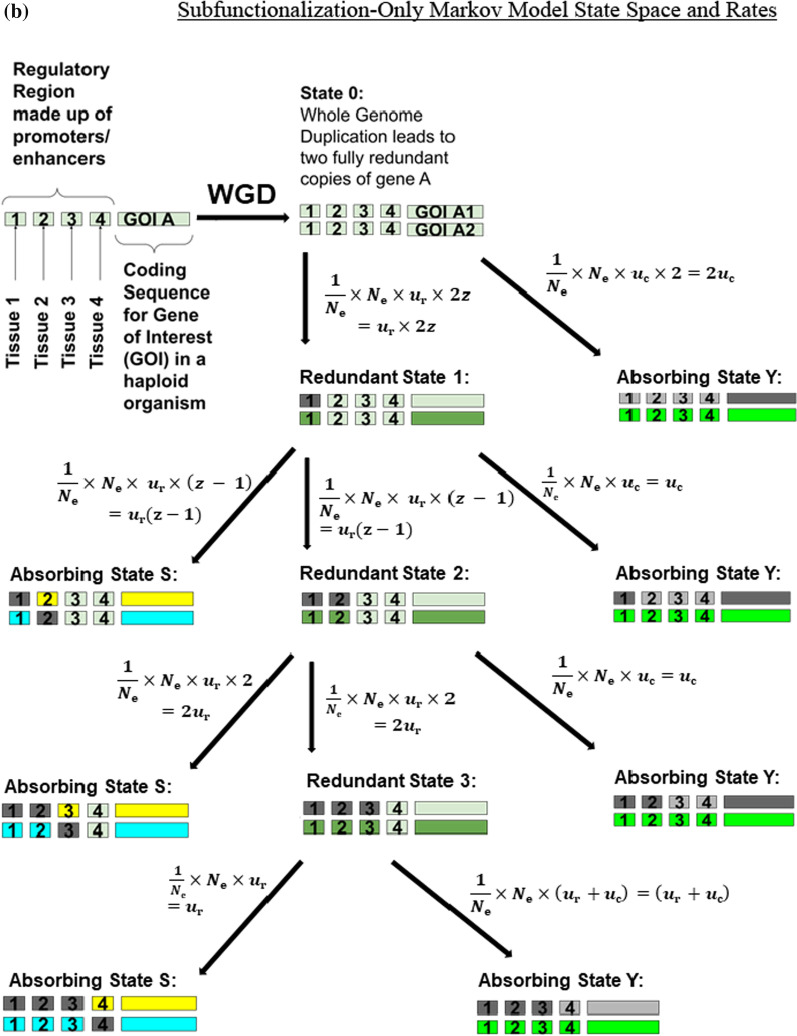
Markov model for the fate of retention of duplicate copies after gene duplication. In this example, the gene of interest (GOI A) has been duplicated into two copies, GOI A_1_ and GOI A_2_. Both copies of GOI A have been duplicated with all four of their regulatory domains upstream of the gene. Each regulatory domain acts as an enhancer for tissues 1–4. The state space includes the two copies with full redundancy (State 0), transient unresolved states (States 1–3), and absorbing states (States Y, S) indicated by neon colors. The absorbing states include nonfunctionalization of one of the gene copies (State Y) and subfunctionalization that leads to the retention of both copies (State S). The neon colors represent which copy is permanently retained and what function(s) it preforms. The light green parts indicate unresolved portions of the gene. The parts of the gene that are dark grey indicate that part being knocked-out through mutation. The parts of the gene that are light grey indicate parts of the gene that are no longer functional because of mutations that occurred in other parts of the gene. Note that for both part a and b, the rate equation for State 1 → State S is equal to the rate equation for State 1 → State 2 AND the rate equation for State 2 → State S is equal to the rate equation for State 2 → State 3. **a** Subfunctionalization + Dosage Model. The formulas for the rates between states are calculated using a fixation probability equation [66], which uses the relative fitness of the current state (*f*_*i*_) and the next state (*f*_*j*_), and the effective population size (*N*_e_). The rates also incorporate the number of regulatory domains (*z*), the nucleotide length of the regulatory domains (*l*_r_), the nucleotide length of the coding region (*l*_c_), the loss of function nucleotide mutation rate (*u*_b_, *u*_h_). **b** Subfunctionalization-Only Model [59] for the fate of retention of duplicate copies after gene duplication. The formulas for the rates between states are calculated the effective population size (*N*_e_), the number of regulatory domains (*z*), Poisson rate at which null mutations are fixed in each of the *z* mutable regulatory regions for each gene (*u*_r_, Eq. 10), and the Poisson rate at which null mutations fix in the coding regions (*u*_c_, Eq. 9)

We define a continuous-time Markov chain $\left\{\mathrm{X}\left(\mathrm{t}\right),\mathrm{t}\ge 0\right\} \left(\mathrm{Figure }1\mathrm{a}\right)$ for our Subfunctionalization + Dosage (Sub + Dos) Model that is similar to that of the Stark et al. [59] model we refer to as, the Subfunctionalization-Only (Sub-Only) Model (Fig. 1b). We use the same state space,

8 $$\mathcal{A} =\left\{\mathrm{0,1},\dots ,\mathrm{z}-1\right\}\hspace{0.17em}\cup \hspace{0.17em}\left\{\mathrm{S},\mathrm{Y}\right\},$$

and state $\mathrm{i}\in \{\mathrm{0,1},\dots ,\mathrm{z}-1\}$ each represent a set of duplicate gene pairs where one duplicate copy has that number of nonfunctional regulatory regions. State S represent a duplicate gene pair that has been subfunctionalized and State Y represent a duplicate gene pair where one copy has been lost (pseudogenized). Both State S and Y are absorbing states.

Like that of the Sub-Only Model [59], our Sub + Dos Model is also based on the mechanics of regulatory subfunctionalization, the assumption that knock-out mutations occur at a constant rate and are independent of each other, and that selection ensures that at least one copy of each regulatory region is retained. Also, like Stark et al. [59] model, we assume a haploid genome to avoid the complication of recombination. Results would be at least partially readily extendable to the diploid case with a natural model for the role of dominance resulting from the explicit link with underlying biochemistry. This makes the model more readily extendable from haploid to diploid cases than purely statistical genetics models that are naïve to the underlying biochemical processes that generate patterns of dominance. The model does not deal with recombination between alleles or context-dependent allele behavior in a diploid setting. Duplicates are assumed to be fixed in the genome. It might be noted that during the early phases of duplication in a haploid setting, the duplicates can behave like alleles in a diploid population.

In contrast with the Sub-Only Model [59], our Sub + Dos Model incorporates dosage balance affects by assuming that the process of losing a regulatory function is non-neutral and that the probability of fixing a loss is not simply 1/*N*_e_. Instead, we assume that fitness is inversely proportional to the magnitude of dosage imbalance introduced by the loss. We estimate dosage imbalance stoichiometrically through the concentration of exposed hydrophobic residues ([hp]) (Eq. 6). Therefore, the relative fitness of each state of duplicate gene pairs is inversely proportional to the sum of [hp] across expression domains.

We use the fitness of each state, that incorporates the fitness penalty associated with loss of expression, to calculate the probability of fixation [66] (Eq. 7) and use that to calculate the rate of transition between states (equation set 11). Because the fitness of each state is only affected by the sum of [hp], the probability of fixing a loss mutation is therefore determined by the concentration of exposed hydrophobic residues that loss introduces. We chose to model the effect of dosage balance in this way because is consistent with expected underlying mechanisms [26, 67].

Additionally, we expanded the parameters set from those in Stark et al. [59] for the Sub-Only model to better reflect empirical data. We conceptualize the Poisson rate at which null mutations fix in the coding regions (*u*_c_) and the Poisson rate at which null mutations are fixed in each of the *z* mutable regulatory regions for each gene (*u*_r_) as being calculated by specific types of mutations that lead to the loss of function and the opportunity for those mutations that can be empirically measured. We calculate the rate of loss of a regulatory domain (*u*_r_) as the product of the nucleotide rate of certain mutations that impair transcription factor binding (*u*_b_) and the nucleotide length of regulatory regions (*l*_r_) (Eq. 10). We calculate the rate of loss of the coding region (*u*_c_) as the product of nucleotide rate of mutations that lead to a non-functional mRNA or peptide chain (*u*_h_) and the nucleotide length of coding region (*l*_c_) (Eq. 9). However, in this paper we do not explore the effects of changing the rates of mutations that impair transcription factor binding and those that lead to a nonfunctional protein for simplicity. Instead, we make the reasonable assumption that both rates of loss of function would be caused by similar types of mutations, including but not limited to mutations that directly affect transcription factor binding and mutations that affect the phasing of DNA binding sites, therefore having comparable nucleotide mutation rates. However, in application of our model, these rates can easily be different from those employed here.

9 $$u_{{\text{c}}} = (u_{{\text{h}}} \cdot l_{{\text{c}}} \cdot N_{{\text{e}}} )/N_{{\text{e}}} = u_{{\text{h}}} \cdot l_{{\text{c}}}$$

10 $$u_{{\text{r}}} = (u_{{\text{b}}} \cdot l_{{\text{r}}} \cdot N_{{\text{e}}} )/N_{{\text{e}}} = u_{{\text{b}}} \cdot l_{{\text{r}}}$$

Therefore, the generator matrix for the Subfunctionalization + Dosage Markov Chain is defined to be Q = [*q*_*ij*_], where the matrix form of Q is shown in Table 1. The non-zero off-diagonals are given by *q*_*ij*_ (equation set 11). A defense for these transition rates can be found in Stark et al. [59] in conjunction with our above argument on the changes. Because Q is the generator/transition rate matrix, the rows sum to 0. To accomplish this, all other *i,j*^th^ entries are 0 except the diagonals, which are zero minus the sum off the defined row terms. Again, where S and Y are absorbing states with S referring to the Subfunctionalization State, and Y referring to the Pseudogenization/Nonfunctionalization state.

**Table 1:** The Q Matrix form for the new Subfunctionalization + Dosage Model, given in equation set 10

	0	1	2	3	S	Y
0	$- \frac{{1 - \left( {\frac{{f_{0} }}{{f_{1} }}} \right)}}{{1 - \left( {\frac{{f_{0} }}{{f_{1} }}} \right)^{{N_{e} }} }} \cdot N_{e} \cdot u_{b} \cdot l_{r} \cdot 2z$ $- 2*\frac{{1 - \left( {\frac{{f_{0} }}{{f_{{\text{Y}}} }}} \right)}}{{1 - \left( {\frac{{f_{0} }}{{f_{{\text{Y}}} }}} \right)^{{N_{e} }} }} \cdot N_{e} \cdot u_{h} \cdot l_{c}$	$\frac{{1 - \left( {\frac{{f_{0} }}{{f_{1} }}} \right)}}{{1 - \left( {\frac{{f_{0} }}{{f_{1} }}} \right)^{{N_{e} }} }} \cdot N_{e} \cdot u_{b} \cdot l_{r} \cdot 2z$	$0$	$0$	$0$	$2* \frac{{1 - \left( {\frac{{f_{0} }}{{f_{{\text{Y}}} }}} \right)}}{{1 - \left( {\frac{{f_{0} }}{{f_{{\text{Y}}} }}} \right)^{{N_{e} }} }} \cdot {N}_{\mathrm{e}}\cdot {u}_{\mathrm{h}}\cdot {l}_{\mathrm{c}}$
1	$0$	$- \frac{{1 - \left( {\frac{{f_{1} }}{{f_{2} }}} \right)}}{{1 - \left( {\frac{{f_{1} }}{{f_{2} }}} \right)^{{N_{e} }} }} \cdot N_{e} \cdot u_{b} \cdot l_{r} \cdot (z - 1)$ $- \frac{{1 - \left( {\frac{{f_{1} }}{{f_{{\text{S}}} }}} \right)}}{{1 - \left( {\frac{{f_{1} }}{{f_{{\text{S}}} }}} \right)^{{N_{e} }} }} \cdot N_{e} \cdot u_{b} \cdot l_{r} \cdot (z - 1)$ $- \frac{{1 - \left( {\frac{{f_{1} }}{{f_{{\text{Y}}} }}} \right)}}{{1 - \left( {\frac{{f_{1} }}{{f_{{\text{Y}}} }}} \right)^{{N_{e} }} }} \cdot N_{e} \cdot u_{h} \cdot l_{c}$	$\frac{{1 - \left( {\frac{{f_{1} }}{{f_{{\text{2}}} }}} \right)}}{{1 - \left( {\frac{{f_{1} }}{{f_{{\text{2}}} }}} \right)^{{N_{e} }} }} \cdot {N}_{\mathrm{e}}\cdot {u}_{\mathrm{b}}\cdot {l}_{\mathrm{r}}\cdot (z-1)$	$0$	$\frac{{1 - \left( {\frac{{f_{1} }}{{f_{{\text{S}}} }}} \right)}}{{1 - \left( {\frac{{f_{1} }}{{f_{{\text{S}}} }}} \right)^{{N_{e} }} }} \cdot {N}_{\mathrm{e}}\cdot {u}_{\mathrm{b}}\cdot {l}_{\mathrm{r}}\cdot (z-1)$	$\frac{{1 - \left( {\frac{{f_{1} }}{{f_{{\text{Y}}} }}} \right)}}{{1 - \left( {\frac{{f_{1} }}{{f_{{\text{Y}}} }}} \right)^{{N_{e} }} }} \cdot {N}_{\mathrm{e}}\cdot {u}_{\mathrm{h}}\cdot {l}_{\mathrm{c}}$
2	$0$	$0$	$- \frac{{1 - \left( {\frac{{f_{2} }}{{f_{{\text{3}}} }}} \right)}}{{1 - \left( {\frac{{f_{2} }}{{f_{{\text{3}}} }}} \right)^{{N_{e} }} }} \cdot {N}_{\mathrm{e}}\cdot {u}_{\mathrm{b}}\cdot {l}_{\mathrm{r}}\cdot \left(z-2\right)$ $- \frac{{1 - \left( {\frac{{f_{2} }}{{f_{{\text{S}}} }}} \right)}}{{1 - \left( {\frac{{f_{2} }}{{f_{{\text{S}}} }}} \right)^{{N_{e} }} }} \cdot {N}_{\mathrm{e}}\cdot {u}_{\mathrm{b}}\cdot {l}_{\mathrm{r}}\cdot \left(z-2\right)$ $- \frac{{1 - \left( {\frac{{f_{2} }}{{f_{{\text{Y}}} }}} \right)}}{{1 - \left( {\frac{{f_{2} }}{{f_{{\text{Y}}} }}} \right)^{{N_{e} }} }} \cdot {N}_{\mathrm{e}}\cdot {u}_{\mathrm{h}}\cdot {l}_{\mathrm{c}}$	$\frac{{1 - \left( {\frac{{f_{2} }}{{f_{{\text{3}}} }}} \right)}}{{1 - \left( {\frac{{f_{2} }}{{f_{{\text{3}}} }}} \right)^{{N_{e} }} }} \cdot {N}_{\mathrm{e}}\cdot {u}_{\mathrm{b}}\cdot {l}_{\mathrm{r}}\cdot (z-2)$	$\frac{{1 - \left( {\frac{{f_{2} }}{{f_{{\text{S}}} }}} \right)}}{{1 - \left( {\frac{{f_{2} }}{{f_{{\text{S}}} }}} \right)^{{N_{e} }} }} \cdot {N}_{\mathrm{e}}\cdot {u}_{\mathrm{b}}\cdot {l}_{\mathrm{r}}\cdot (z-2)$	$\frac{{1 - \left( {\frac{{f_{2} }}{{f_{{\text{Y}}} }}} \right)}}{{1 - \left( {\frac{{f_{2} }}{{f_{{\text{Y}}} }}} \right)^{{N_{e} }} }} \cdot {N}_{\mathrm{e}}\cdot {u}_{\mathrm{h}}\cdot {l}_{\mathrm{c}}$
3	$0$	$0$	$0$	$- \frac{{1 - \left( {\frac{{f_{3} }}{{f_{{\text{S}}} }}} \right)}}{{1 - \left( {\frac{{f_{3} }}{{f_{{\text{S}}} }}} \right)^{{N_{e} }} }} \cdot {N}_{\mathrm{e}}\cdot {u}_{\mathrm{b}}\cdot {l}_{\mathrm{r}}\cdot \left(z-3\right)$ $- \frac{{1 - \left( {\frac{{f_{3} }}{{f_{{\text{Y}}} }}} \right)}}{{1 - \left( {\frac{{f_{3} }}{{f_{{\text{Y}}} }}} \right)^{{N_{e} }} }} \cdot {N}_{\mathrm{e}}\cdot {u}_{\mathrm{h}}\cdot {l}_{\mathrm{c}}$ $- \frac{{1 - \left( {\frac{{f_{3} }}{{f_{{\text{Y}}} }}} \right)}}{{1 - \left( {\frac{{f_{3} }}{{f_{{\text{Y}}} }}} \right)^{{N_{e} }} }} \cdot {N}_{\mathrm{e}}\cdot {u}_{\mathrm{b}}\cdot {l}_{\mathrm{r}}$	$\frac{{1 - \left( {\frac{{f_{3} }}{{f_{{\text{S}}} }}} \right)}}{{1 - \left( {\frac{{f_{3} }}{{f_{{\text{S}}} }}} \right)^{{N_{e} }} }} \cdot {N}_{\mathrm{e}}\cdot {u}_{\mathrm{b}}\cdot {l}_{\mathrm{r}}\cdot (z-3)$	$\frac{{1 - \left( {\frac{{f_{3} }}{{f_{{\text{Y}}} }}} \right)}}{{1 - \left( {\frac{{f_{3} }}{{f_{{\text{Y}}} }}} \right)^{{N_{e} }} }} \cdot {N}_{\mathrm{e}}\cdot {u}_{\mathrm{h}}\cdot {l}_{\mathrm{c}}$ + $\frac{{1 - \left( {\frac{{f_{3} }}{{f_{{\text{Y}}} }}} \right)}}{{1 - \left( {\frac{{f_{3} }}{{f_{{\text{Y}}} }}} \right)^{{N_{e} }} }} \cdot {N}_{\mathrm{e}}\cdot {u}_{\mathrm{b}}\cdot {l}_{\mathrm{r}}$
S	$0$	$0$	$0$	$0$	$0$	$0$
Y	$0$	$0$	$0$	$0$	$0$	$0$

Model parameters and variables are defined in Table 2. Equations to calculate the fitness parameter (*f*) can be found in Eq. 6

**Table 2:** The list of symbols and definitions for parameters and variables, which are used and/or defined in Eqs. 1–11

Parameters and Variables	Symbol	Realistic range based on empirical data	Range used in provided figures
Poisson rate of nucleotide mutations that interfere with transcription binding	*u*_b_	1.0 × 10^–8^–2.5 × 10^–8^ nucleotide mutations per generation [70]	2.5 × 10^–8^ nucleotide mutations per generation
Poisson rate of nucleotide mutations that interfere with production of a functional peptide sequence	*u*_h_	1.0 × 10^–8^–2.5 × 10^–8^ nucleotide mutations per generation [70]	2.5 × 10^–8^ nucleotide mutations per generation
Length of coding region	*l*_c_	5.0 × 10^4^ nucleotides [71]	5.0 × 10^4^ nucleotides
Length of enhancer	*l*_r_	50 bp to 1.5 kbp [72]	775 nucleotides
Subfunctionalization-Only Model poisson rate at which null mutations fix in the coding regions [59]	*u*_c_	–	Equation 9, loss of function mutations per generation
Subfunctionalization-Only Model poisson rate at which null mutations are fixed in each of the *z* mutable regulatory regions for each gene [59]	*u*_r_	–	Equation 10, loss of function mutations per generation
Number of regulatory regions/domains (promoters/enhancers/silencers)	*z*	< 20 regulatory regions	4 regulatory regions
Effective population size	*N*_e_	1.4 × 10^4^ individuals	1 × 10^2^–1.4 × 10^6^ individuals
Time in generations since duplication event	*t*	5.0 × 10^3^–1.4 × 10^4^generations	5.0 × 10^3^–1.0 × 10^4^generations
Equilibrium constant	*K*_eq_	1.0 × 10^6^–1.0 × 10^14^ mol/mL [73, 74]	1.0 × 10^4^–1.0 × 10^12^ mol/mL
Scalar on the relationship between the number of hydrophobic patches per cell and the corresponding fitness penalty	*w*	–	1.0
Heterodimer of interest	AB	–	Equation 1
Gene that codes for subunit A in heterodimer AB	G_A_	–	–
Gene that codes for subunit B in heterodimer AB	G_B_	–	–
Subunit of heterodimer of interest, gene product of G_A_	A	–	Equation 1
Subunit of heterodimer of interest, gene product of G_B_	B	–	Equation 1
Concentration of the heterodimer of interest that is in its bound form	[AB]	–	
Total concentration of subunit A, likely to be estimated by transcription data	[A]_total_	1 × 10^–6^–1 × 10^–10^ mol/mL [75, 76]	2.5 × 10^–6^ mol/mL
Total concentration of subunit B, gene product of G_B_, likely to be estimated by transcription data	[B]_total_	1 × 10^–6^–1 × 10^–10^ mol/mL [75, 76]	2.5 × 10^–6^ mol/mL
Concentration of subunit A in the unbound form	[A]_free_	–	Equation 3, in mol/mL
Concentration of subunit B in the unbound form	[B]_free_	–	Equation 4, in mol/mL
Concentration of exposed hydrophobic patches	[hp]	–	Equation 2, in mol/mL
Current state	i	–	–
Next possible state	j	–	–
Fitness of state	*f*	–	Equation 6
Fitness of current state	*f*_i_	1.0	Equation 7
Fitness of next possible state	*f*_j_	–	Equation 7
Probability of fixation of mutation	*g*	–	Equation 7 [66]
Subfunctionalization, an absorbing state	S	–	Equations 8 and 11
Pseudogenization/nonfunctionalization, an absorbing state	Y	–	Equations 8 and 11

Potential ranges for each parameter are listed, as well as the range used in the production of the figures provided

11 $${q}_{ij}=\left\{\begin{array}{l}2*{g}_{0,Y}*{N}_{\mathrm{e}}*{u}_{\mathrm{h}}*{l}_{\mathrm{c}}, \quad if \;\; i = 0, j = Y\\ 2*z*{g}_{\mathrm{0,1}}*{N}_{\mathrm{e}}*{u}_{b}*{l}_{\mathrm{r}}, \quad if \, i=0, j=1\\ \begin{array}{cc}{g}_{i,Y}*{N}_{\mathrm{e}}*{u}_{\mathrm{h}}*{l}_{\mathrm{c}},& \quad if \; 1\le i\le z-2, j=Y\end{array}\\ \begin{array}{cc}\left(z-i\right)*{g}_{i,j}*{N}_{\mathrm{e}}*{u}_{\mathrm{b}}*{l}_{\mathrm{r}},& \end{array}if \,1\le i\le z-2, j=i+1\\ \begin{array}{cc}\left(z-i\right)*{g}_{i,S}*{N}_{\mathrm{e}}*{u}_{\mathrm{b}}*{l}_{\mathrm{r}},& \quad if 1\le i\le z-2, j=S\end{array}\\ {g}_{z-1,Y}*{N}_{\mathrm{e}}*{u}_{\mathrm{b}}*{l}_{\mathrm{r}}+g*{N}_{\mathrm{e}}*\begin{array}{cc}{u}_{\mathrm{h}}*{l}_{\mathrm{c}},& \quad if \; i=\end{array}z-1, j=Y\\ \begin{array}{cc}{g}_{z-1,Y}*{N}_{\mathrm{e}}*{u}_{\mathrm{b}}*{l}_{\mathrm{r}},& \quad if \; i=z-1, j=S.\end{array}\end{array}\right.$$

We define the probability matrix as P = [*p*_*ij*_]. For each generation, P is calculated by exponentiating *e* to the product of time in the number of generations and Q.

### Probability distribution calculations

The computer program, written in C++, can be found at https://github.com/aewilson96/Wilson_Liberles_2022, calculates the probability distribution of states for a pair of genes for each generation following a whole-genome duplication event. Because of the nature of Markov Chains, we can directly calculate the probability distribution from the generator matrix; therefore, there is no need to run simulations over time. The calculation for the rate of transitioning from one state to the other, includes the probability of fixation (*g*, Eq. 7), the effective population size (*N*_e_), the nucleotide rate of loss of function mutations (*u*_b_ and *u*_h_), the length of the coding sequence (*l*_c_), and the length of the regulatory domain (*l*_r_, enhancer, promoter, silencer). The probability of fixation calculation [66] utilizes the relative fitness of the current state (*f*_*i*_) and the relative fitness of the next possible state (*f*_*j*_). To determine the relative fitness of each state, our method assumes an inverse relationship between the summation of the concentration of exposed hydrophobic patches summed across expression domains and fitness (Eq. 6). Using the equilibrium constant (*K*_eq_), the concentration of hydrophobic residues ([hp]) is calculated for when the total concentration of gene A products and gene B products are in stoichiometric balance, and for when they are in a 1:2 ratio, which is the expected imbalance for a pair of gene homologs if one copy is not functionally expressed (Eq. 6). Then, for each state, these values are summed across expression domains, being the summation of both (1) the product of unaffected domains and the concentration of hydrophobic patches when they are in stoichiometric balance and (2) the product of the affected domains and the concentration of hydrophobic patches when they are stoichiometrically imbalanced.

Small-scale duplication events work a little differently than whole-genome duplication events. These events cause immediate stochiometric imbalance and losing a copy of the duplicated gene is expected to repair the balance. Because of this difference, the concentration of hydrophobic residues is expected to be greater immediately after the duplication event, and nonfunctionalization/pseudogenization becomes the state with the highest fitness because it repairs the stoichiometric balance quickest, therefore having the highest probability of fixing. Additionally, losing redundancy and subfunctionalizing are still more favorable states than the totally redundant State 0. This is different than in the whole-genome duplication case, because for whole-genome duplication events, losing expression in anyway yields a lower fitness (because of the higher concentration of hydrophobic residues); therefore, these dosage effects slow progression through the states towards the absorbing states. In small-scale duplication events, this progression is faster when dosage balance effects act.

**Fig. 2:**
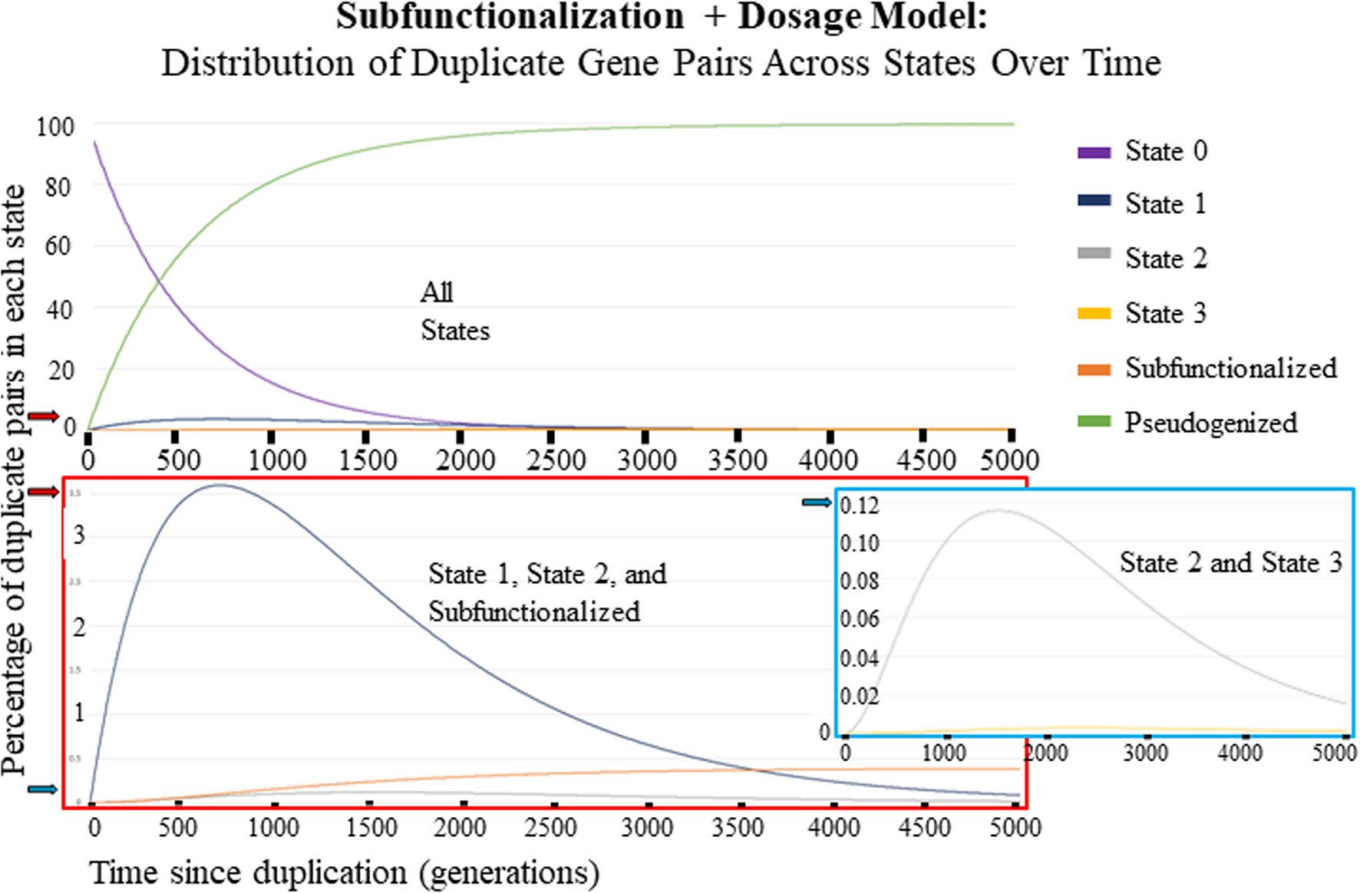
Distribution of Duplicate Gene Pairs across States over 5000 generations, represented as a percentage. The purple line is the percentage of gene pairs that are completely redundant. The dark blue line is the percentage of gene pairs in State 1 with one of the copies having lost one expression domain. The gray line is the percentage of gene pairs in State 2 with one of the copies having lost two expression domains. The yellow line is the percentage of gene pairs in State 2 with one of the copies having lost three of four expression domains. The orange line is the percentage of gene pairs where the expression domains have been subfunctionalized. The green line is the percentage of gene pairs where one of the copies have been completely nonfunctionalized/pseudogenized. The red box is a zoomed in graph of the percentage of gene pairs in State 1, State 2 and Subfunctionalized. The blue box is a zoomed in graph of the percentage of gene pairs in State 2 and State 3. The red arrow indicates where 3.5% is on the y axis. The blue arrow indicates where 0.12% is on the y axis

**Fig. 3:**
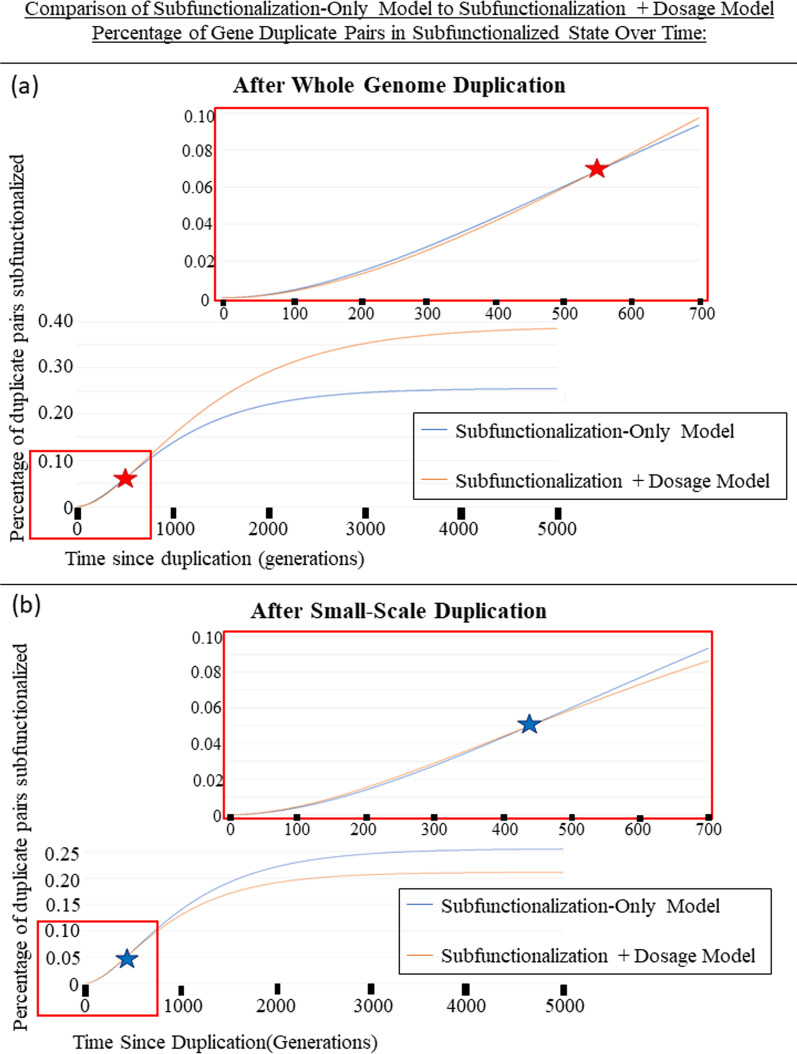
**a** The percentage of gene pairs that have been subfunctionalized over 5000 generations after a Whole-genome Duplication Event. The blue line is the Subfunctionalization-Only Model. The orange line is the new Subfunctionalization + Dosage Model. The red box shows the graph zoomed in to 700 generations and 0.1% gene pairs. The red star represents where the two lines cross, prior to the star, Sub-Only Model has a higher percentage of gene pairs that are subfunctionalized, while after the star, the Sub + Dos model has a higher percentage of gene pairs that have been subfunctionalized. **b** The percentage of gene pairs that have been subfunctionalized over 5000 generations after a Small-Scale Duplication Event. The blue line is the Subfunctionalization-Only Model. The orange line is the new Subfunctionalization + Dosage Model. The red box shows the graph zoomed in to 700 generations and 0.1% gene pairs. The blue star represents where the two lines cross, prior to the star, Sub-Only Model has a lower percentage of gene pairs that are subfunctionalized, while after the star, the Sub + Dos model has a lower percentage of gene pairs that have been subfunctionalized

**Fig. 4:**
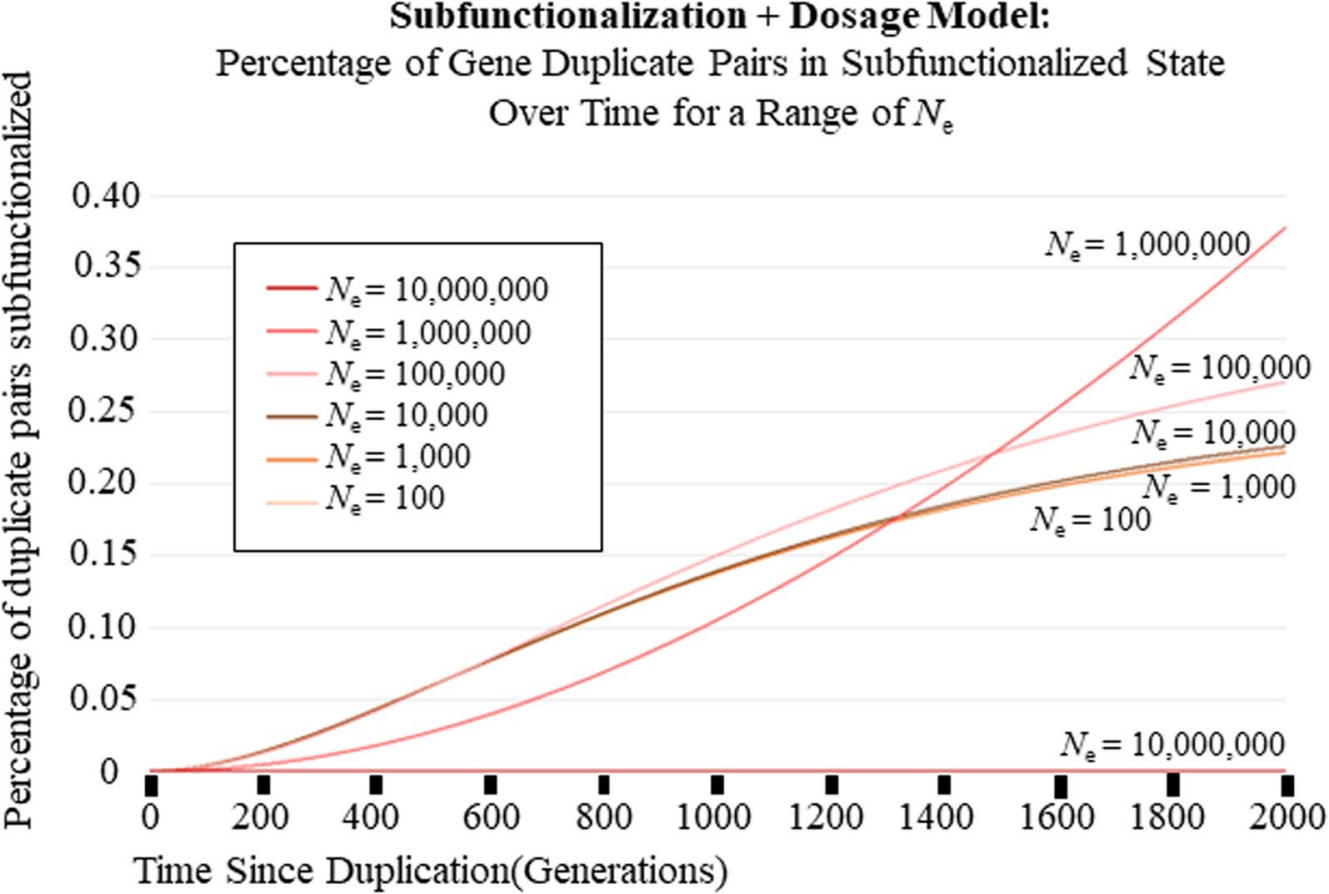
The percentage of gene pairs that have been subfunctionalized over 2000 generations after a whole-genome Duplication Event for 6 different effective population sizes (*N*_e_) for the new Sub + Dos Model. Note that as the effective population size increases, so does the efficacy of selection, and that leads to the pattern where there is a longer delay for subfunctionalization to occur, but will ultimately lead to a higher percentage of subfunctionalized duplicate gene pairs. Also note that with the largest *N*_e_ shown is so delayed, that it hasn’t even begun to subfunctionalize for the number of generations shown, however will ultimately surpass the others in the percentage of subfunctionalized gene pairs

**Fig. 5:**
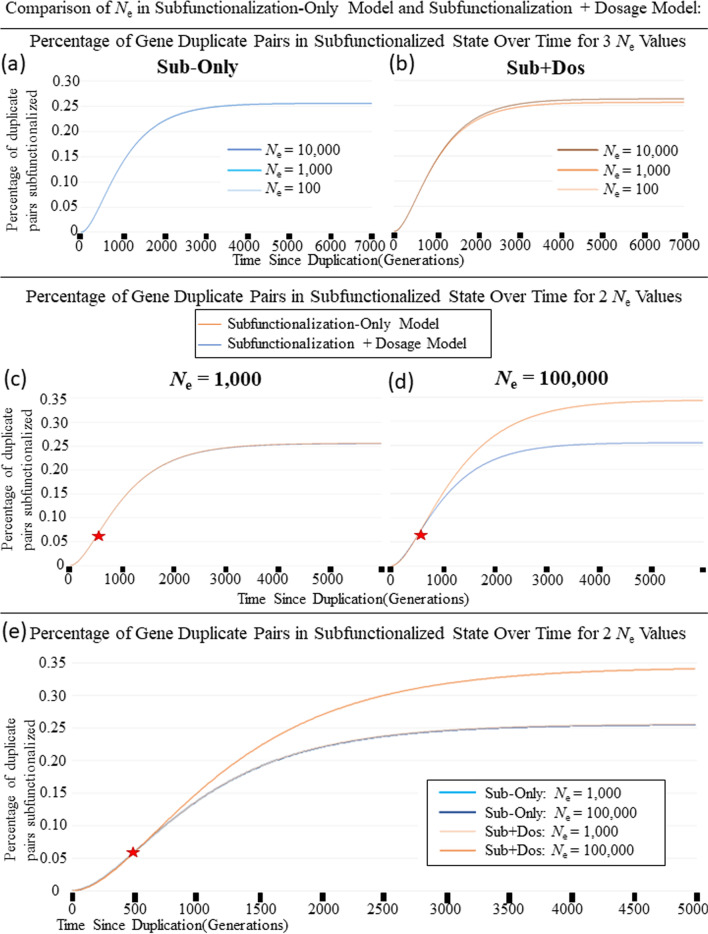
A comparison of the effect that *N*_e_ has on the resulting percentage of gene pairs that have subfunctionalized after a whole-genome duplication event for the Subfunctionalization-Only Model and the Subfunctionalization + Dosage Model. The blue lines are the Subfunctionalization-Only Model and the orange lines are the Subfunctionalization + Dosage Model (**a**) Subfunctionalization-Only Model over 7000 generations for 3 different effective population sizes (*N*_e_). Note that the percentage of subfunctionalized genes is the same at any given time because the effective population size does not affect the rate of subfunctionalization in this model. **b** Over 7000 generations for 3 different effective population sizes (*N*_e_) for our Subfunctionalization + Dosage Model. Note that as the effective population size increases, so does the efficacy of selection, and that leads to the pattern where there is a longer delay for subfunctionalization to occur, but will ultimately lead to a higher percentage of subfunctionalized duplicate gene pairs. **c** Over 6000 generations comparing the Subfunctionalization-Only Model to our Subfunctionalization + Dosage Model with effective population sizes (*N*_e_) = 1000. The red star represents where the two lines cross, prior to the star, Sub-Only Model has a higher percentage of gene pairs that are subfunctionalized, while after the star, our Sub + Dos model has a higher percentage of gene pairs that have been subfunctionalized. **d** Over 6000 generations comparing the Subfunctionalization-Only Model to our Subfunctionalization + Dosage Model with effective population sizes (*N*_e_) = 100,000. The red star represents where the two lines cross, prior to the star, Sub-Only Model has a higher percentage of gene pairs that are subfunctionalized, while after the star, our Sub + Dos model has a higher percentage of gene pairs that have been subfunctionalized. **e** Over 5000 generations comparing the Subfunctionalization-Only Model to our Subfunctionalization + Dosage Model for two effective population sizes (*N*_e_) = 1000 (lighter colored lines) and *N*_e_ = 100,000 (darker colored lines). For each *N*_e_ value, initially the Sub-Only model has a higher percentage of gene pairs that are subfunctionalized, but eventually our Sub + Dos Model has a higher percentage of gene pairs that have been subfunctionalized

**Fig. 6:**
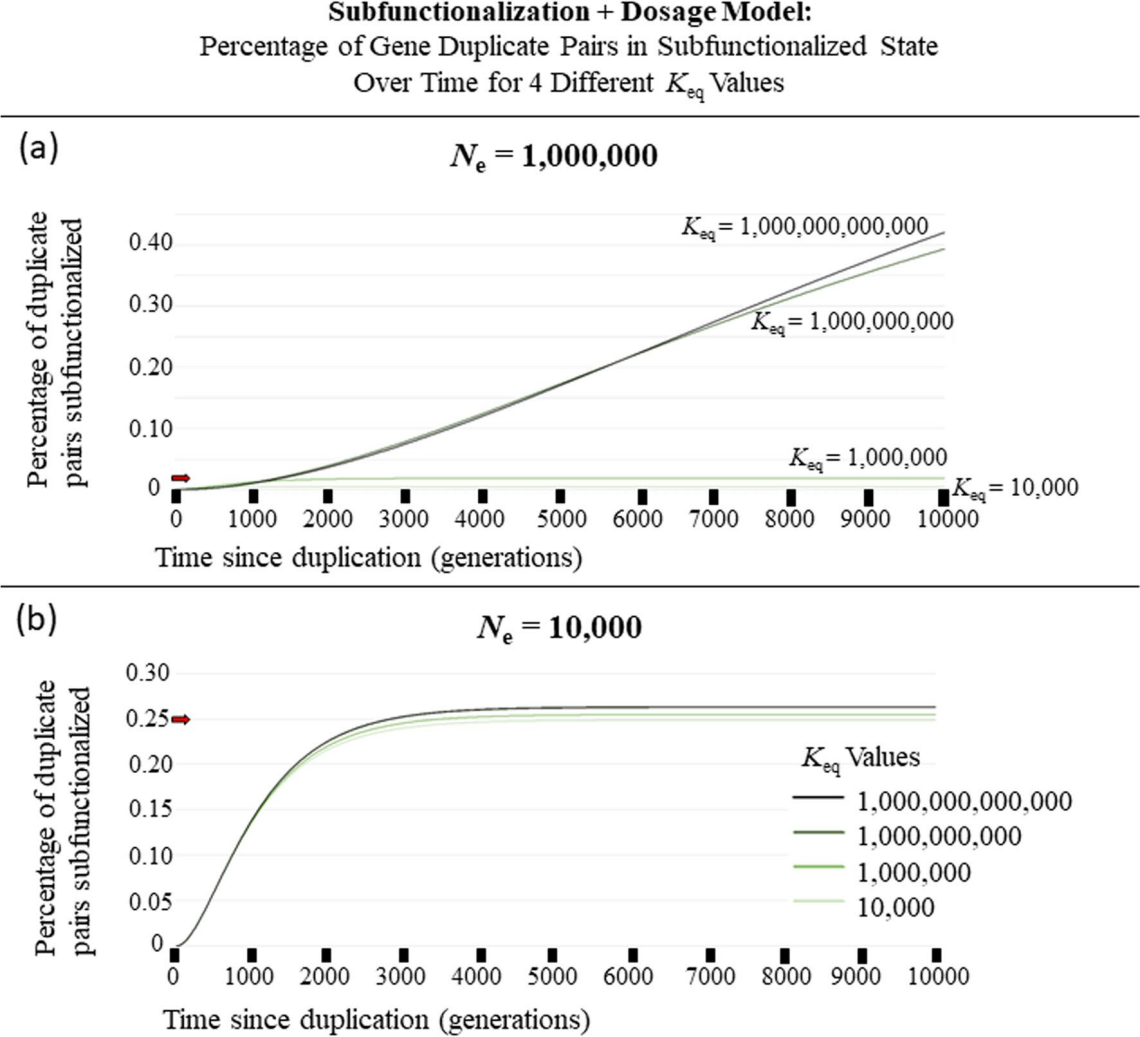
The percentage of gene pairs that have been subfunctionalized over 10,000 generations with an effective population size (*N*_e_) of **a** 1,000,000 **b** 10,000 after a Whole-genome Duplication Event for 4 different equilibrium constants (*K*_eq_) for our Subfunctionalization + Dosage Model. Note that as *K*_eq_ increases, so does the strength of selection, and that leads to the pattern where there is a longer delay for subfunctionalization to occur but will ultimately lead to a higher percentage of subfunctionalized duplicate gene pairs

All of the figures that show the Subfunctionalization + Dosage (Sub + Dos) Model have 4 regulatory regions/domains (*z*), a scalar of 1.0 (*w*), a nucleotide mutation rate that affects transcription of a functional coding strand of 2.5 × 10^–8^ nucleotide mutations per generation (*u*_h_), a nucleotide mutation rate that affects transcription binding to regulatory region/domain of 2.5 × 10^–8^ nucleotide mutations per generation (*u*_b_), 5.0 × 10^4^ nucleotide long coding regions, and 775 nucleotide long regulatory region/domains. The equivalent parameters used for all figures that show the Subfunctionalization-Only (Sub-Only)[59] Model is 4 regulatory regions/domains (*z*), 1.25 × 10^–3^ mutations per generation that affects transcription binding to regulatory region/domain(*u*_r_ = *u*_b_ ⋅ *l*_c_), 1.9375 × 10^–5^ mutations per generation that affect transcription of a functional coding strand (*u*_c_ = *u*_h_ ⋅ *l*_r_). Figures 2, 3, 4, and 5 all used a *K*_eq_ value of 1.0 × 10^10^ mol/mL. Figure 6 used a range of *K*_eq_ values including 1.0 × 10^4^; 1.0 × 10^6^; 1.0 × 10^9^; 1.0 × 10^12^ (mol/mL). For all figures, the concentration of total A was 2.5 × 10^–6^ mol/mL immediately after duplication. The same is true for concentration of total B, except for Fig. 3b, because that models a small-scale duplication event, where B was not duplicated so the concentration of total B used was 1.25 × 10^–6^ mol/mL. The figures show anywhere between 2.0 × 10^3^ and 1.0 × 10^4^ generations after the duplication event, chosen based on figure clarity. The effective population size chose for each figure was 1.4 × 10^5^ individuals for Figs. 2 and 3. For Figs. 4, 5a–e and 6, we used a range of *N*_e_’s from as low as 100 to as much as 1.0 × 10^7^.

All computer software is available on Github at https://github.com/aewilson96/Wilson_Liberles_2022.

### Expected impact of parameter choices

We wanted to use parameter values that existed in a realistic range in order to show the magnitude of the effect that dosage balance has on the rate of subfunctionalization. To obtain biologically realistic values, we conducted a literature search for values that were equal to our parameters, or a proxy that would be on a similar order of magnitude as our parameters (see Tables 2 and 3). We chose these values to perform our calculations because they represented close proxies and had good evidence for them; however, it is expected that the general behavior shown in the results section holds true regardless of the values used. The logical argument for this expectation is as follows.

**Table 3:** Parameters that change for the Subfunctionalization + Dosage Model for each figure

Figure number	*K*_eq_ (mol/mL)	*N*_e_ (individuals)	Time (generations)	[A]_total_ immediately after duplication (mol/mL)	[B]_total_ immediately after duplication (mol/mL)
2	10,000,000,000	140,000	5000	0.0000025	0.0000025
3a	10,000,000,000	140,000	5000 (zoomed in 700)	0.0000025	0.0000025
3b (Small-Scale)	10,000,000,000	140,000	5000 (zoomed in 700)	0.0000025	0.00000125
4	10,000,000,000	100; 1,000; 10,000; 100,000; 1,000,000; 10,000,000	2000	0.0000025	0.0000025
5a (Sub-Only)	10,000,000,000	100; 1,000; 10,000	7000	0.0000025	0.0000025
5b (Sub + Dos)	10,000,000,000	100; 1,000; 10,000	7000	0.0000025	0.0000025
5c	10,000,000,000	100,000; 1,000	6000	0.0000025	0.0000025
5d	10,000,000,000	100,000; 1,000	6000	0.0000025	0.0000025
5e	10,000,000,000	1,000; 100,000	5000	0.0000025	0.0000025
6	10,000; 1,000,000; 1,000,000,000; 1,000,000,000,000	10,000; 1,000,000	10,000	0.0000025	0.0000025
	10,000,000,000	100; 1,000; 10,000; 100,000; 1,000,000; 10,000,000	2000	0.0000025	0.0000025

The Subfunctionalization-Only Model shares the same time parameter for the corresponding figure

The* z* values are equal for the rate calculations for both the Subfunctionalization-Only Model [59]and Subfunctionalization + Dosage Model. As shown in Eqs. 9 and 10, *u*_c_ is equivalent to *u*_h_ ⋅* l*_c_, and *u*_r_ is equivalent to *u*_b_ ⋅ *l*_r_. Additionally, *u*_h_, *l*_c_, *u*_b_, and *l*_r_ > 0, because the mutation rate would always be positive, and the length of the regions will be positive. Therefore, *u*_c_ and *u*_r_ > 0.

So, we can ignore *u*_c_ and *u*_r_ from Sub-Only Model and *u*_h_ ⋅ *l*_c_ and *u*_b_ ⋅ *l*_r_ from Sub + Dos Model because they are equal. The only difference in the rate calculation of the Sub + Dos Model from the Sub-Only Model is *g* ⋅ *N*_e_ equals 1 in the Sub-Only equation (because *N*_e_ ⋅ 1/*N*_e_ = 1), but Sub + Dos calculates *g* as the probability of fixation from the relative fitnesses of each state [66]. So therefore, the important factor is how the behavior of rate = 1 is different than *g* ⋅ *N*_e_.

Because in whole-genome duplication events, [hp] will increase to some extent with the introduction of more imbalance, even if it is fractionally small, the fitness of the next state will always be lower than that of the current state. A number larger than 1 to the *N*_e_^th^ power, given the *N*_e_^th^ power is larger than 1 (which is a reasonable assumption for population sizes), will also be larger than 1. Therefore, the rate of transitioning to the next state will always be less than 1 for the Sub + Dos Model, given these assumptions, so it will always have a smaller rate than the rate in the Sub-Only Model. Note that having more introduced imbalance will always make the fitness of that state lower relatively, so nonfunctionalization will be less favorable than subfunctionalization for *z* ≥ 2, so the rate of nonfunctionalization will be even lower than the rate of subfunctionalization in the Sub + Dos Model.

Therefore, we should expect to see the same pattern presented by our results after a whole-genome duplication events for parameters that abide by our assumptions. To summarize, these assumptions include that the nucleotide mutation rate is positive, the length of the nucleotide regions is positive, subfunctionalization and nonfunctionalization are neutral in the Sub-Only model, subfunctionalization and nonfunctionalization receive a fitness penalty associated with the extent of imbalance the state introduces, and the population size is greater than 1.

## Results

Figure 1a shows the state space and transition rates for the Subfunctionalization + Dosage-Balance Model. Figure 1b shows the Subfunctionalization-Only Model taken from Stark et al. [59], which has the same state space, but different transition rates. There are several similarities between the transition rate calculations for these two models. One similarity is that they both have the same opportunity for a new mutation to occur (mutation rates) because the state space is the same. The main difference is how the probability of fixation is calculated. The Sub-Only model combines several parameters into one parameter, with the probability of fixing a mutation (*u*_r_ or *u*_c_) as being the product of the allele frequency in the population (1/*N*_e_), the population size (*N*_e_), the rate of nucleotide mutation rate (*u*_b_ or *u*_h_), and the length of the region in question (*l*_r_ or *l*_c_). Because there is one allele per individual as both models assume haploidy, 1/*N*_e_ and *N*_e_ cancel out and are therefore not included in their rate calculations. Because of the cancelation of parameters, the Sub-Only Model’s *u*_r_ and *u*_c_ can be directly compared to the product of the nucleotide mutation rate (*u*_b_ or *u*_h_), and the length of the region in question (*l*_r_ or *l*_c_) in the Sub + Dos Model. Therefore, for the purpose of comparison, we assumed their *u*_r_ to be equal to *u*_b_ ⋅ *l*_r_ and *u*_c_ to be equal to *u*_h_ ⋅ *l*_c_. This expansion of parameter space was necessary to properly mirror the complexity of the biological processes involved in dosage compensation and allows us to input empirical information into the rate equations. While the Sub + Dos is also a haploid model, it uses the relative fitnesses of each state to calculate the probability of fixation [66]; therefore these values are included in the rate calculations. These fitnesses are correlated with the total concentration of hydrophobic residues, which are a representation of the amount of imbalance each state introduces between interacting partners.

**Fig. 7:**
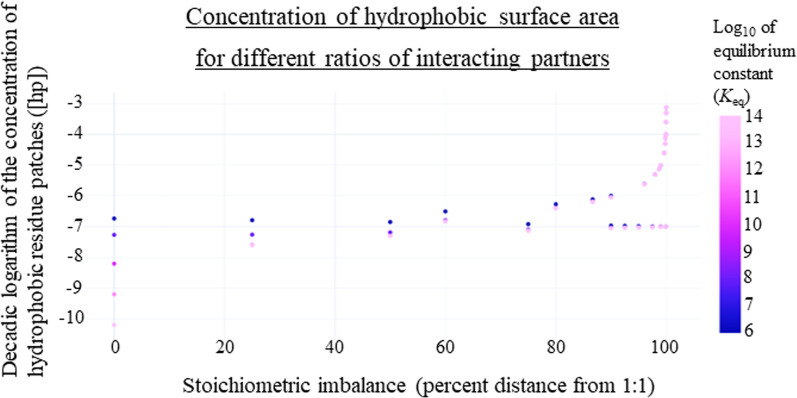
Concentration of hydrophobic surface area for different ratios of interacting partners. Concentration of hydrophobic surface area is shown on a log scale, as well as equilibrium constant values (*K*_eq_). When the *K*_eq_ values are larger, and the ratio of interacting partners ([A]_total_/[B]_total_) is closer to one, the sum of [A]_free_ and [B]_free_ is smaller. Similarly, when the *K*_eq_ values are smaller, and the ratio of interacting partners ([A]_total_/[B]_total_) is further from one, the sum of [A]_free_ and [B]_free_ is larger. This is possibly explained by the fact that overall exposed hydrophobic patches would be lower if A and B are in balance and are tight binders

Our results show that with more imbalanced interacting partners, there is an increased concentration of cellular exposed hydrophobic patches, which would lead to more spurious deleterious reactions occurring (Fig. 7). In addition, we found that with smaller *K*_eq_, we also see this increased concentration of exposed hydrophobic patches because of the lower affinity, leading to more gene products to exist in the unbound state, also leading to more spurious deleterious interactions (Fig. 7). Also notably, the more imbalanced the partners are, the bigger the difference is between [hp] values with a small versus large *K*_eq_. For Fig. 7, stoichiometric imbalance is presented as a percent distance away from the 1:1 ratio, where the ratios were scaled where 0% was a 1:1 ratio and 100% imbalanced is the ratio with the largest magnitude, therefore the furthest from 1:1. If [A]_total_ ≥ [B]_total_, we used [B]_total_/[A]_total_ as the ratio and if [B]_total_ > [A]_total_ then, we used [A]_total_/[B]_total_ as the ratio.

The parameters used for the Sub + Dos Model across all figures are 4 regulatory regions/domains (*z*), fitness scalar of 1.0 (*w*), 2.5 × 10^–8^ nucleotide mutations per generation (*u*_h_ and *u*_b_), coding region 50,000 nucleotides long (*l*_c_), and regulatory region 775 nucleotides long (*l*_r_). The equivalent parameters used for the Sub-Only Model across all figures are 4 regulatory regions/domains (*z*), 1.25 × 10^–3^ coding region mutations per generation (*u*_r_ = *u*_b_ ⋅ *l*_c_), and 1.9375 × 10^–5^ regulatory region mutations per generation (*u*_c_ = *u*_h_ ⋅ *l*_r_). Table 3 lists the parameters that are not the same throughout all the figures. Figure 2 shows the percentage of gene pairs in each state over 5000 generations for the Sub + Dos Markov model using parameter values found in Table 1. The Markov model begins in a fully redundant state and two absorbing states are possible, nonfunctionalization and subfunctionalization. As expected, under this modeling framework using these parameters, > 99% of redundant genes nonfunctionalize and a small fraction subfunctionalize.

Figure 3a shows 5,000 generations after a whole-genome duplication event. When using equivalent parameters, the initial rate of subfunctionalization parameters is higher with the Sub-Only Model than in the new Sub + Dos Model after a whole-genome duplication event. However, as indicated by a star, a transition occurs and the equilibrium level of subfunctionalization is much higher with the Sub + Dos model. Table 4 shows the frequencies of different states over time under the different models.

**Table 4:** Comparison of the resulting percentage of genes in each state for Sub-Only Model and Sub + Dos Model is shown below for 400 Generations and 4,000,000 Generations following whole-genome duplication

Percentage of genes in each state	400 Generations Sub-Only	4,000,000 Generations Sub-Only	400 Generations Sub + Dos	4,000,000 Generations Sub + Dos
State 1: fully redundant (transient)	34.58%	0%	52.19%	0%
State 2: Loss of 1 enhancer (transient)	2.805%	0%	3.283%	0%
State 3: Loss of 2 enhancers on one copy (transient)	0.03559%	0%	0.03486%	0%
State 4: Loss of 3 enhancers on one copy (transient)	0.0001927%	0%	0.0001603%	0%
State S: Subfunctionalized (absorbing)	0.04339%	0.2557%	0.04053%	0.4687%
State Y: One copy pseudogenized (absorbing)	62.54%	99.74%	44.46%	99.53%

Alternatively, Fig. 3b shows 5000 generations after a small-scale duplication event. It shows how the comparison of the behaviors between models would change after a small-scale duplication. Again, here we show that the initial subfunctionalization rate is faster with the Sub + Dos model, but is eventually overtaken by the Sub-Only model, which is in fact, the opposite as it is after a whole-genome duplication event. This pattern is intuitive because dosage balance is initially preserved after a whole-genome duplication event, but not after a small-scale event, making the fitness of losing a mutation after a small-scale event more favorable.

The results obtained in Fig. 2 are dependent upon choices of parameters (Table 3). A range of parameter values exploring the effects of *N*_e_ (and thereby selection) (Fig. 5b–e, Additional file 1: Fig. S1) and of equilibrium binding constants (Fig. 6) were explored. Figure 4, shows how the Sub + Dos model changes for 6 different *N*_e_ values between 100 and 1.0 × 10^7^ individuals over 2000 generations. These results show that as the *N*_e_ increases, so does the efficacy of selection, resulting in a longer delay for subfunctionalization to occur but eventually leading to more subfunctionalized gene pairs, and this holds true when compared to the Sub-Only model (Fig. 5b–e, Additional file 1: Fig. S1). This effect, a longer delay but ultimately more subfunctionalized pairs, was also observed for increasing binding affinity.

## Discussion

When dosage balance effects are added to a model for subfunctionalization, this leads to increased retention after whole-genome duplication events after an initial delay in the rate of subfunctionalization. Consistent with observations on differential retention patterns for smaller-scale duplication, the opposite trends are observed, with a reduction in the probability of terminal subfunctionalization.

It should be noted that while most duplicate genes are lost and are lost relatively quickly from genomes, this process is slowed down in whole-genome duplication events relative to small-scale duplication events in genomic data [3, 30] and is slowed down for duplicates that are dosage balanced [55, 68, 69]. The model has tunable parameters for the selective strength that will affect the absolute levels of retention, but the qualitative effects are observable over broad ranges of parameterization.

Dosage balance as a process generates a time-dependent selective barrier to subfunctionalization and to nonfunctionalization. The dynamics of this process involve delayed terminal subfunctionalization, but subfunctionalization at higher rates in the end. Because this is selective and is dependent upon *N*_e_, it emerges that subfunctionalization of genes when dosage balance processes are acting is not a purely neutral process. This is a finding that has not previously been described in the literature to our knowledge.

While we have not independently varied *w*, the selective scalar, this becomes convoluted with *N*_e_ in determining selective effects and variation in *w* would be expected to mirror variation in *N*_e_. Variation in *K*_eq_ reveals that higher *K*_eq_ values, that favor subunits in their bound form, also leads to increased delay but higher rates of terminal subfunctionalization, because it increases the selection against imbalanced proteins because fewer subunits are in the unbound state when in balance.

In this study, we have assumed that gene expression levels of each duplicate remain constant. Ascencio et al. [49] found that in fact and as expected, gene expression evolves as a co-evolutionary process after gene duplication. While this was not modeled for the sake of simplicity, changing gene expression as a stochastic process could be added to the model to examine the dynamics under those scenarios. Further, the interactions that were modeled were those that reflected a heterodimer that forms a stable interaction. The extension to trimers and higher order heterocomplexes is possible, and is expected to yield similar results, but no longer enables simple analytical transition probabilities of the type described here. Additionally, the main findings are expected to hold true for any gene that is sensitive to dosage balance effects. Another simplifying assumption was the expression in only two tissues. Stark et al. [59] explored the role of the number of tissues expressed in the dynamics of subfunctionalization without dosage; that complexity could also be ported over to this model, with clear expectations of the resulting dynamics. An increase in the number of independent tissue expressions increases the rate of nonfunctionalization relative to subfunctionalization and therefore would be expected to increase the selective effect of the dosage barrier.

Another component that has been ignored to date is the deus ex machina process of neofunctionalization. Adding neofunctionalization, depending upon the associated assumptions about functional redundancy, has the potential to change the dynamics described here. The addition of neofunctionalization will create a fuller mechanistic model for duplicate gene fates, but is beyond the scope of the study here. There is complexity in identifying reasonable assumptions for the mutational process leading to neofunctionalization, which is why this is not as straight forward as it might appear.

While other levels of biological complexity in the underlying population genetics and molecular evolutionary processes are conceivable to model (including *trans-*effects), it is important to recognize the change in dynamics and process associated with just adding dosage balance to the characterization of the subfunctionalization process.

## Conclusions

The complex dynamics of the interplay between subfunctionalization and dosage balance leads to opposite expectations for the timing and probabilities of retention for genes that encode proteins that function as multimeric complexes compared to those that function as monomers or homomultimers between whole-genome duplication and smaller-scale duplication. For proteins that function in multimeric complexes, retention following whole-genome duplication events through subfunctionalization is expected to be a non-neutral process.

## Supplementary Information

**Additional file 1**: Figure S1.

## Data Availability

The computer programs in the C++ programming language used for the analysis here are available on github through the following link: https://github.com/aewilson96/Wilson_Liberles_2022.

## Abbreviations

SSD: Small-scale duplication
WGD: Whole genome duplication
*K*_eq_: Equilibrium constant
*N*_e_: Effective population size
Sub + Dos: Subfunctionalization + dosage model
Sub-Only: Subfunctionalization only model from Stark et al. [59]
