# Supplementary material for: Dosage balance acts as a time-dependent selective barrier to subfunctionalization
Source: BMC Ecol Evol. 2023 May 3;23:14. doi: 10.1186/s12862-023-02116-y (PMC10155369; doi:10.1186/s12862-023-02116-y)
Supplement: Supplementary file 1 — Additional file 1: Figure S1. [file 12862_2023_2116_MOESM1_ESM.docx]

Wilson and Liberles, Supplemental Materials

*
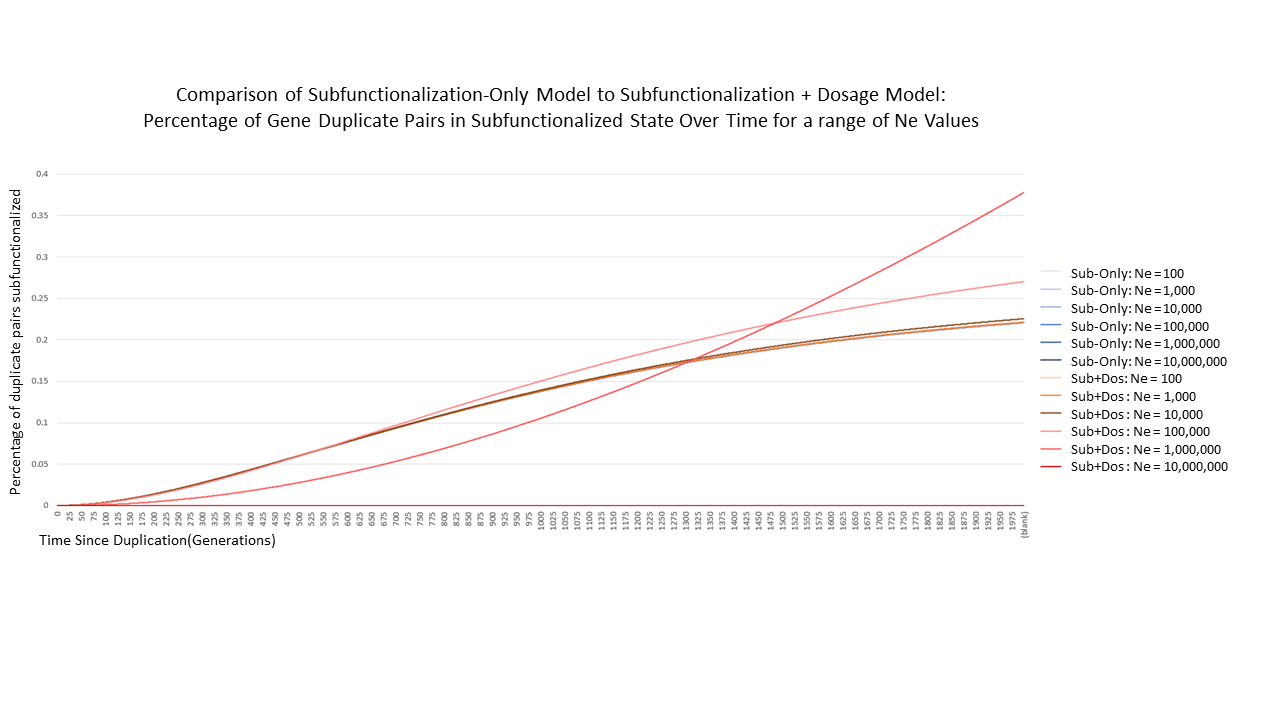
*

Supplemental Figure 1: The percentage of gene pairs that have been subfunctionalized over 2000 generations after a Whole Genome Duplication Event comparing the Subfunctionalization-Only model to our Subfunctionalization+Dosage Model for six effective population sizes. The blue lines are the Sub-Only Model. The lighter the blue line, the smaller the Ne, and the darker the blue line the larger the Ne. The orange lines are the Sub+Dos model. The lighter the orange line, the smaller the N_e_, and the darker the orange line the larger the N_e_. For each Ne value, initially the Sub-Only model has a higher percentage of gene pairs that are subfunctionalized, but eventually our Sub+Dos model has a higher percentage of gene pairs that have been subfunctionalized. Note that for our Sub+Dos Model, as the effective population size increases, so does the efficacy of selection, and that leads to the pattern where there is a longer delay for subfunctionalization to occur, but will ultimately lead to a higher percentage of subfunctionalized duplicate gene pairs. Also note that for Sub-Only Model, the percentage of subfunctionalized genes is the same at any given time because the effective population size does not affect the rate of subfunctionalization in this model.
